# *QuickStats:* Age-Adjusted Death Rates,* by State — United States,^^†^^ 2017

**DOI:** 10.15585/mmwr.mm6810a7

**Published:** 2019-03-15

**Figure:**
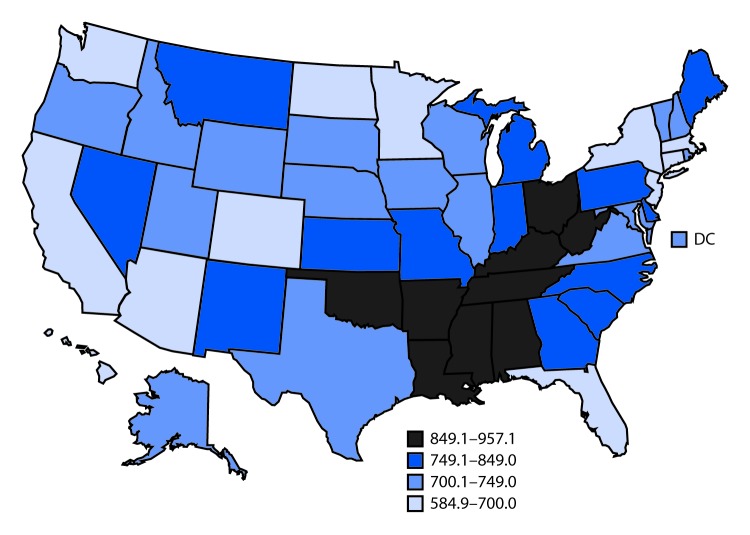
In 2017, the overall U.S. death rate was 731.9 per 100,000 standard population; rates varied by state. The five states with the highest age-adjusted death rates were West Virginia (957.1 deaths per 100,000 standard population), Mississippi (951.3), Kentucky (929.9), Alabama (917.7), and Oklahoma (902.4). The five states with the lowest death rates were Hawaii (584.9), California (618.7), New York (623.6), Connecticut (651.2), and Minnesota (656.4).

**Source:** National Vital Statistics System. Underlying cause of death data, 1999–2017. https://wonder.cdc.gov/ucd-icd10.html.

